# Veronal in the Vomiting of Pregnancy1Translation from Therapeutische Monatshefte, 1908. No. 7.

**Published:** 1910-03

**Authors:** A. Reich, A. Herzfeld

**Affiliations:** New York; New York


### ABSTRACT

---

### Veronal in the Vomiting of Pregnancy[1]

By A. REICH, M. D. and A. HERZFELD, M. D., New York.

The number of drugs which have been recommended for the treatment of vomiting of pregnancy is large, and we are well aware that the favorable mention of a new one does not signify that a specific has been discovered. On the other hand, the favorable results which we have obtained with veronal in the treatment of this affection stamp it as a valuable remedy and will justify this article.

We have been in a position to follow up sixteen cases to the normal termination of pregnancy, and a number are still under observation. Veronal seemed to do most good where there was no other cause for the vomiting but the pregnancy itself (the so-called reflex vomiting of nervous and excitable women).

The following cases deserve mention:

Case IV.—H. B., 25 years old, 3-para. Patient was always well, family history negative. The woman was very excitable and inclined to be hysterical. Abortion had been induced twice in the third month, owing to vomiting which could not be checked. After the last interference Dr. Reich found a retroverted uterus which could easily be brought back into the right position and kept there with a Hodge-Smith pessary. The patient was very anxious to have a family, and again became pregnant. At the end of the second month intractable vomiting again set in, on account of which the pessary was removed. The condition did

1. Translation from Therapeutische Monatshefte, 1908, No. 7.

not improve, and the vomiting continued unabated, despite all medication. The family physician decided to again induce abortion. At this time the patient complained of severe burning pains in the epigastrium, and constant salivation. The gums were red and bled easily, and other symptoms were a dry tongue, fetor from the mouth, constipation, and a decided odor of acetone on the breath. The patient had lost much weight.

Dr. Reich advised that 3 grn. of veronal be given every three hours, in hot water, for two days. The vomiting stopped at once. Magnesia usta was ordered later for the constipation. There was no more emesis for a month; when it reappeared it was again checked by giving 2½ grn. of veronal three times a day, in hot water. In the fourth month it was necessary to repeat the medication for two days, with the same result. After this there was no recurrence. Some time later the patient gave birth to a healthy child.

Case V.—L. B., primipara, 23 years old, suffering from hystero-epilepsy, for which she was treated with the bromides. Severe vomiting set in during the sixth week of pregnancy, which persisted for two weeks before the first dose of 4 grn. of veronal in hot water was given. The patient took 15 grn. daily for two days. The vomiting ceased and did not recur during the pregnancy.

Case VIII.—E. W., 27 years old, 2-para. The patient was formerly a teacher, and was always well, but very excitable and "nervous." Soon after conception there was frequent and severe vomiting. All kinds of drugs were tried. After 2 grn. of veronal three times a day, in hot water, the emesis was checked immediately, and did not recur throughout the entire pregnancy.

Case IX.—H. A., primipara, 35 years old, and the sister of above patient. Soon after conception frequent and severe vomiting set in, which was checked, after the second day, by 2 grn. of veronal every three hours, in hot water. There was no recurrence up to term.

Case XII.—Ch. S., 35 years old, 2-para. The patient suffering formerly from oöphoritis, and is easily excited. During the first pregnancy there was severe vomiting, so that the patient was nourished per rectum for some time. The only food which was retained on the stomach was panopepton. During the first pregnancy she claims to have lost as much as 40 pounds. During the second pregnancy vomiting began in the second month, and was so severe that all food was brought up. Up to the third month panopepton was again given, but the emesis continued. Then 3 grn. of veronal in hot water three times a day was ordered. The insomnia disappeared and the severe vomiting improved, so that meat taken at breakfast was retained. In this case the vomiting could not be checked completely with veronal, since she would frequently eject the noon and evening meal, but never the breakfast. Pregnancy took a normal course, and the woman was easily and quickly confined.

The remaining cases were not severe, and resembled cases VIII and IX. Veronal had a favorable influence upon the emesis in every instance. Veronal is also of service in obstetrical cases, where a hypnotic is indicated, especially in *prolonged labor,* during the period of dilatation, where a somnifacient has a stimulating action. The labor pains are not diminished in intensity, but the pain is less keenly felt. The patients are confined in a semi-stupor. If an operative interference should be required, it is surprising how little anesthetic will be necessary to induce complete narcosis. In such cases 4 to 8 grn. of veronal are given. Disagreeable after-effects have never been seen.

